# Immune Privilege: The Microbiome and Uveitis

**DOI:** 10.3389/fimmu.2020.608377

**Published:** 2021-01-25

**Authors:** Christine Mölzer, Jarmila Heissigerova, Heather M. Wilson, Lucia Kuffova, John V. Forrester

**Affiliations:** ^1^ Institute of Medical Sciences, University of Aberdeen, Aberdeen, United Kingdom; ^2^ Department of Ophthalmology, First Faculty of Medicine, Charles University and General University Hospital in Prague, Prague, Czechia; ^3^ Eye Clinic, Aberdeen Royal Infirmary, Aberdeen, United Kingdom

**Keywords:** T regulatory cells, folate, probiotics, blood retinal barrier, adjuvant effect, commensals, nutritional metabolites

## Abstract

Immune privilege (IP), a term introduced to explain the unpredicted acceptance of allogeneic grafts by the eye and the brain, is considered a unique property of these tissues. However, immune responses are modified by the tissue in which they occur, most of which possess IP to some degree. The eye therefore displays a spectrum of IP because it comprises several tissues. IP as originally conceived can only apply to the retina as it contains few tissue-resident bone-marrow derived myeloid cells and is immunologically shielded by a sophisticated barrier – an inner vascular and an outer epithelial barrier at the retinal pigment epithelium. The vascular barrier comprises the vascular endothelium and the glia limitans. Immune cells do not cross the blood-retinal barrier (BRB) despite two-way transport of interstitial fluid, governed by tissue oncotic pressure. The BRB, and the blood-brain barrier (BBB) mature in the neonatal period under signals from the expanding microbiome and by 18 months are fully established. However, the adult eye is susceptible to intraocular inflammation (uveitis; frequency ~200/100,000 population). Uveitis involving the retinal parenchyma (posterior uveitis, PU) breaches IP, while IP is essentially irrelevant in inflammation involving the ocular chambers, uveal tract and ocular coats (anterior/intermediate uveitis/sclerouveitis, AU). Infections cause ~50% cases of AU and PU but infection may also underlie the pathogenesis of immune-mediated “non-infectious” uveitis. Dysbiosis accompanies the commonest form, HLA-B27–associated AU, while latent infections underlie BRB breakdown in PU. This review considers the pathogenesis of uveitis in the context of IP, infection, environment, and the microbiome.

## Introduction

**Figure 1:**
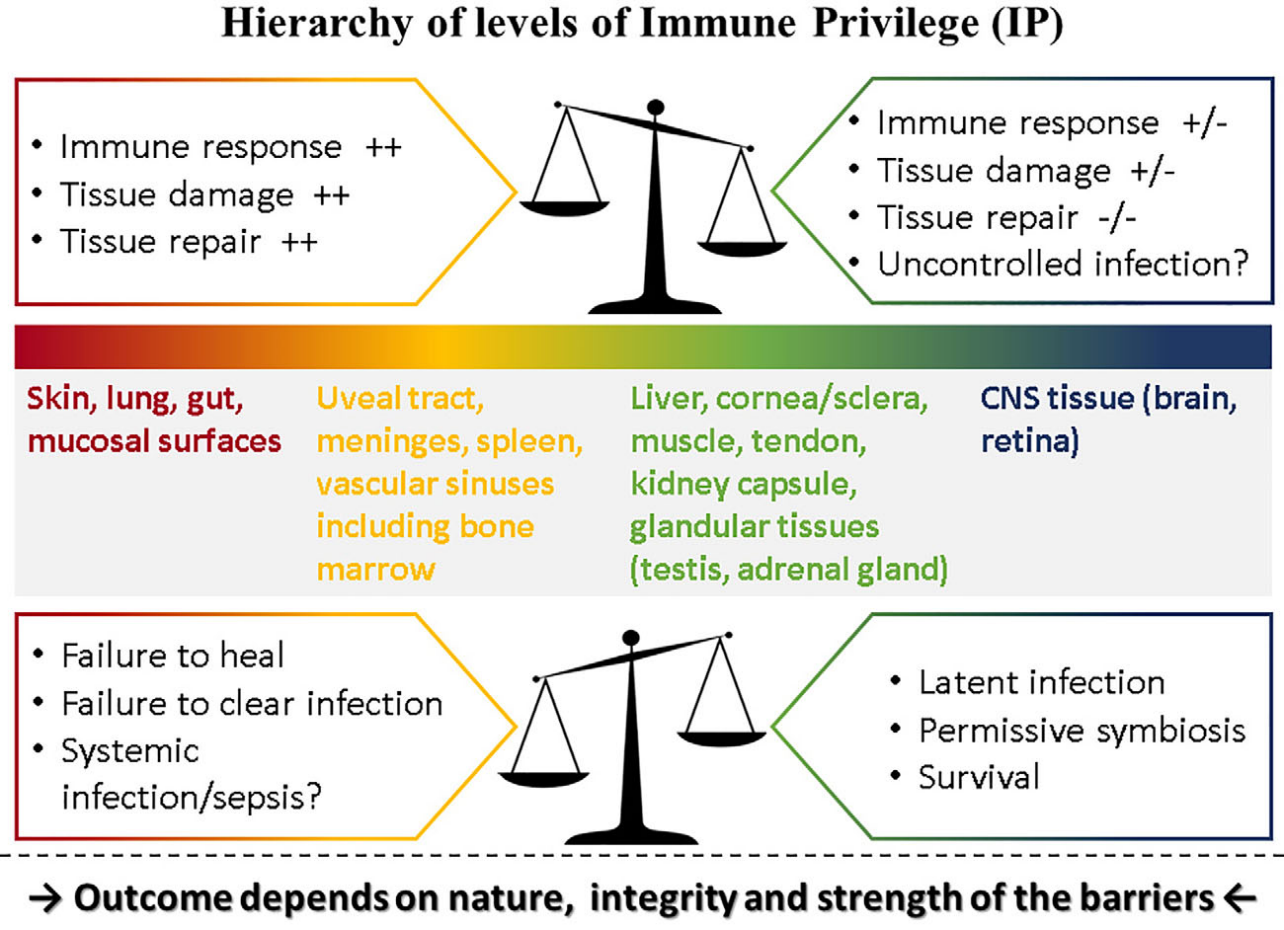
Hierarchy of levels of Immune Privilege (IP). IP may be considered as a property of all tissues with varying degrees of ability to influence the outcome of immune responses played out within their own microenvironment. Thus, tissues such as the skin and mucosal surfaces generate strong immune reactions to pathogens, experience extensive tissue damage and have considerable capacity for repair, while at the other extreme neural tissue (brain parenchyma, retina) temper or even prevent immune responses, induce latency rather than replication in pathogens [reviewed in (3).] but have limited ability for repair if severely damaged. Between these two extremes, different tissues have different levels of “privilege.” The outcome of pathogen challenge to any tissue depends on the level of privilege which is determined by the nature, integrity and strength of that tissue’s barrier to pathogen invasion. Barriers are complex entities comprising physical, chemical, molecular, cellular, and immunological components and are specific to each tissue. The figure illustrates what happens under normal circumstances in the top panel: here the balance of IP is set toward strong immune and repair processes in the skin at one end of the spectrum but a weak immune response in the CNS at the other end of the spectrum risks uncontrolled infection. In contrast, when this balance is shifted as shown in the lower panel, outcomes tend to reverse: in the skin, a weaker immune response might fail to clear infections or fail to promote repair while in the CNS better control of infection might be possible but still does not clear pathogens, instead promoting latent infections.

Immune privilege (IP) was conceived as a protective response to immune challenge by tissues with limited capacity for renewal such as the eye and the brain [reviewed in (1)]. Matzinger and Kamala have suggested that all tissues modulate the immune response including frontline tissues such as skin and mucosa (2). If this is valid, then the eye (and the brain) which are composites of several types of tissue, can be considered to express various levels of “privilege.” Thus, the level of IP for each component of the eye and the brain would match the level of IP of corresponding tissues: in the case of the eye, composed as it is of vascular and avascular connective tissues forming a barrier-lined case around neural tissue, several levels of IP probably hold sway, with the retina at the top of the scale (**Figure 1**).

However, it has been observed in several previous reviews that despite high levels of IP, ocular tissues are susceptible to inflammation and infection (1, 4–9). For instance, the long-held reputation for corneal allograft success only truly applies to specific conditions such as keratoconus and certain dystrophies. Once inflamed the cornea loses its IP and becomes “high risk” for rejection with an allograft success rate possibly less than other vascularized solid organs (10). Similarly, the uveal tract and meninges display levels of IP little different from other vascular tissues [reviewed in (11)], and so it is no surprise that the anterior segment is susceptible to inflammation and that anterior uveitis (AU) is the most common brand of uveitis (12). Probably IP in the eye, if it exists as originally conceived, can be applied only to the retina (11). This is not to diminish in any way the unique immunological properties of the retina and the brain parenchyma which are based on physical, cellular, molecular and immunological features setting them apart from other tissues. Nor does it gainsay the influence all tissues have on the immune response (2). Context is everything and a review of these aspects is presented here.

A new facet to immune processes has emerged based on our rapidly evolving appreciation of the influence of the microbiome on development of immunity. The acquisition and maturation of a healthy microbiome during the post-natal period and early infancy shapes the development of the blood-central nervous system (CNS) barrier (13–15) and so the proper establishment of a healthy microbiome is critically linked to the development of a mature immune system which determines susceptibility to CNS infection during this time. This applies not only to the CNS but also to other systems such as the musculoskeletal system. The long-recognized relationship between inflammatory bowel disease (IBD), the spondylo-arthropathies (SpA) and ocular inflammatory disease is now being re-appraised in terms of a dysregulated microbiome (dysbiosis) (16) which in the context of IP raises many questions. This is also discussed.

## Immune Privilege: How the Concept Arose and How it has Blurred Our Vision Concerning the Regulation of the Immune Response

The concept of IP stems back to the nineteenth century [reviewed in (17)] but the term was coined and popularized by Medawar whose experiments showing acceptance of skin allografts in the anterior chamber of the eye supported the notion (18, 19). In essence, IP was considered a special feature of tissues such as the eye and the brain in which the default immune rejection of allografts and alloantigens failed to occur unless the non-rejected tissue developed connecting blood vessels (20). IP has been attributed to sequestration of tissue self-antigens from the immune system behind blood-tissue barriers, due to lack of blood vessels, lack of lymphatic drainage, absence of MHC Class II antigen presenting cells (APC), high concentrations of immunosuppressive tissue immunomodulators, and more recently, the activity of regulatory T cells (Treg) (21). Several reviews of proposed mechanisms underlying IP have been published (5, 7–9, 21–26).

A second phenomenon linked to IP has also been described namely anterior chamber-associated immune deviation (ACAID) (27–29) as has a similar phenomenon in the brain (brain-associated immune deviation, BRAID) (30). Immune deviation is a recognized immunological phenomenon in which antigen-specific immunity, usually in the form of cell-mediated delayed-type hypersensitivity (DTH), is suppressed while being “deviated” toward humoral immunity, usually the generation of IgG2 antibodies. Antigens inoculated into the anterior chamber of experimental animals generated an ACAID response which usually took the form of a reduced DTH response, but an intact complement-fixing IgG2 B cell response, when re-challenged with the same antigen in the skin. As new discoveries in T cell biology were made, ACAID was relabeled to show that the immune deviation was based on a shift from an antigen-specific Th1 response to Th2 (31). However, it was later shown that systemic Th2 responses were also down-regulated after antigen inoculation into the anterior chamber of the eye (32).

How ACAID, and immune deviation more broadly, was induced became a major research focus in the latter half of the twentieth century. Some form of regulatory cell suppressing DTH T effector cells (Teff) had been assumed and CD4^+^ T cells, CD4^+^ Treg, natural killer (NK) cells and B cells as well as other thymus-derived cells such as regulatory NK-T cells have all been shown to be involved [reviewed in (11)]. Perhaps the most detailed mechanism described has been the induction of alloantigen specific CD8^+^ suppressor cells in the spleen through interaction of invariant NK cells with F4/80^+^ macrophages (7). This is a complex process and the details remain obscure. For instance, it is clear that the spleen is undoubtedly involved since splenectomy abrogates ACAID (33, 34). However, there is a three day window before splenectomy becomes ineffective in preventing ACAID and much has been going on in that interval since signals to immune and trafficking cells are of the order of minutes to hours (35–37). In addition, since there are no lymphatics to the spleen but there are well defined lymphatic channels from the anterior chamber to the eye-draining lymph node (38), the precise trafficking of signals and cells between the anterior chamber, the draining lymph node, the blood circulation, the thymus and eventually the spleen are not clear.

**Figure 2:**
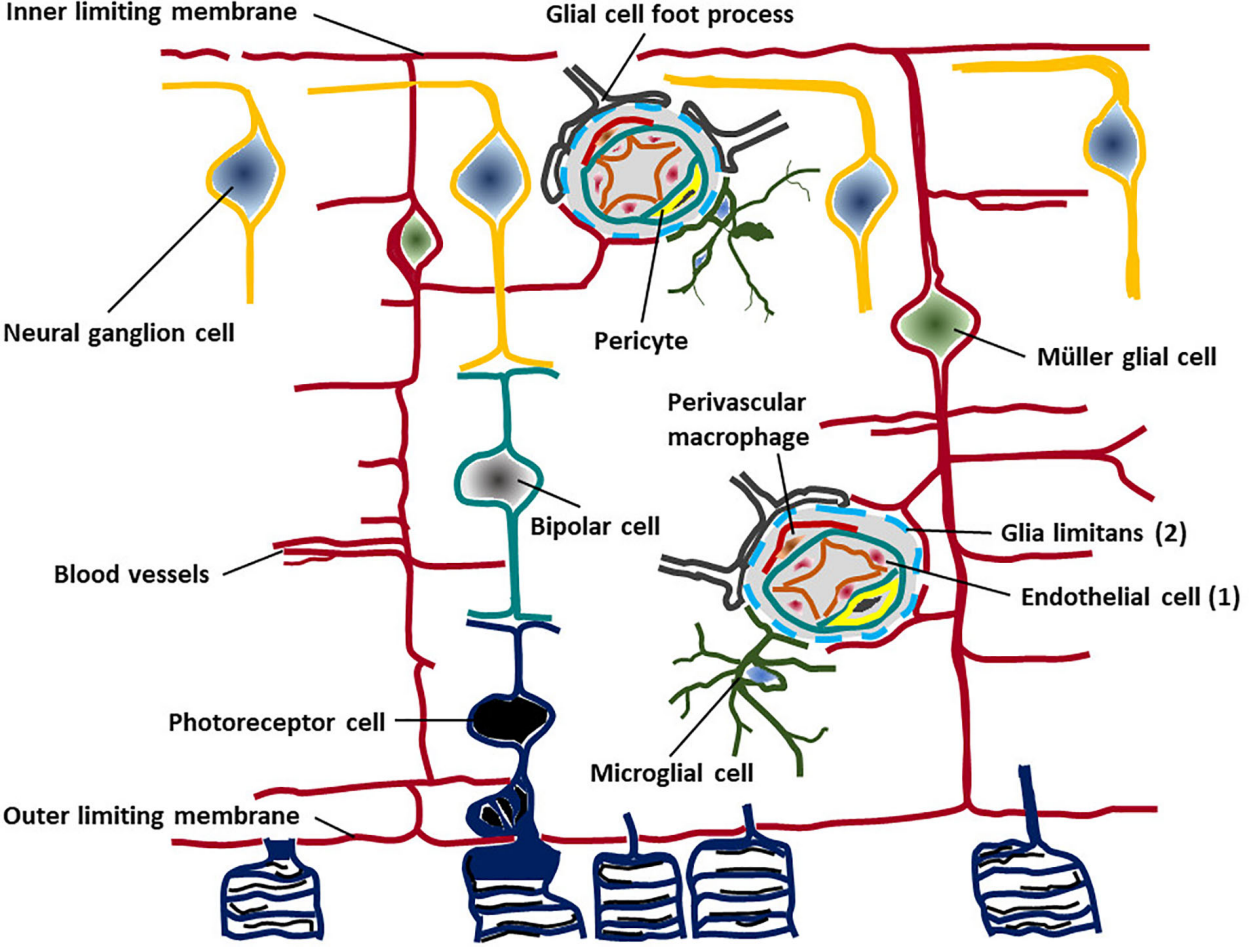
The Retinal Neurovascular Unit. The retinal neurovascular unit (NVU) is the seat of the blood retinal barrier (BRB), situated at the capillary/venous side of the retinal circulation. Two levels of “barrier” exist: a) at the endothelial tight junctions (1), supported by pericytes and under immunological surveillance by perivascular macrophages; and b) at the glia limitans (2), constructed by astrocyte glial cell and Müller glial cell foot processes, with contributions to the continuous membranous structure form microglial cells and neuronal dendritic processes.

There is no denying that ACAID occurs. However, its physiological relevance is less clear. ACAID is an experimental phenomenon and is often equated with IP, but this is not a safe assumption. IP refers to the reduced or modified immune response by tissues due to the properties of that tissue and, as detailed above, is best exhibited by the retina and parenchymal tissue of the brain. ACAID in contrast is a systemic response involving the induction of suppressor/regulatory cells in the periphery, which is revealed by inoculating antigen into the anterior chamber. There is evidence that this systemic phenomenon may be active during homeostasis. Although it has been suggested that CNS parenchymal and retinal antigens are sequestered behind the blood retinal and blood brain barriers (BRB, BBB) (39), i.e. they are “ignored,” recent studies have shown that CNS-specific antigens are recognized by T cells in the periphery but such T cells do not have access to the CNS unless they are activated and do not cause damage except when there is recognition of cognate antigen, e.g. in the context of inflammation with cross-reacting antigens (40, 41). In addition, the BRB (and the BBB) is not only a physical barrier of contiguous endothelial cells bound together by tight junctions, but includes the immunomodulatory pericyte, the resident perivascular macrophage, and the glia limitans composed of the foot processes of glia/Müller cells, with contributions from regulatory microglial cells and neuronal dendrites, all of which comprises the neurovascular unit (NVU) (**Figure 2**). However, the NVU and the BRB/BBB are also presided over by Treg [reviewed in (9, 42)] which theoretically could be the equivalent of ACAID-inducing T suppressor cells and in some sense at least should be specific for CNS antigens, i.e. they should be “educated” by CNS antigen in the way described above.

Whether Treg, which some authors envisage as contributing to the immunological component of the BRB/BBB (43, 44), are physiologically equivalent to the T suppressors and Treg of ACAID may be a question which remains unanswered. More important perhaps is understanding why the BRB and the BBB which normally exclude Teff cells, can be breached if the conditions are right (45). This seems to involve innate immune cells which act as escorts in assisting passage of T cells and further activate T cells when they are *in situ* [reviewed in (46)].

There is considerable evidence that immune protection of the retina (aka IP) involves regulatory immune cells of which there are several types. Treg have a prominent place in overall immune tolerance and are also active in retinal homeostasis. Whether this amounts to a specialized form of immune protection or “privilege” or is a variation on the theme of immunological tolerance (depending on the tissue) may be a fine point. The important issue is to understand how this protection might be lost, particularly if the target of attack is self-antigen. These processes are discussed in the following sections.

## Immune Defense, Immune Privilege, and Tissue Barriers: Internal and External Barriers

**Figure 3:**
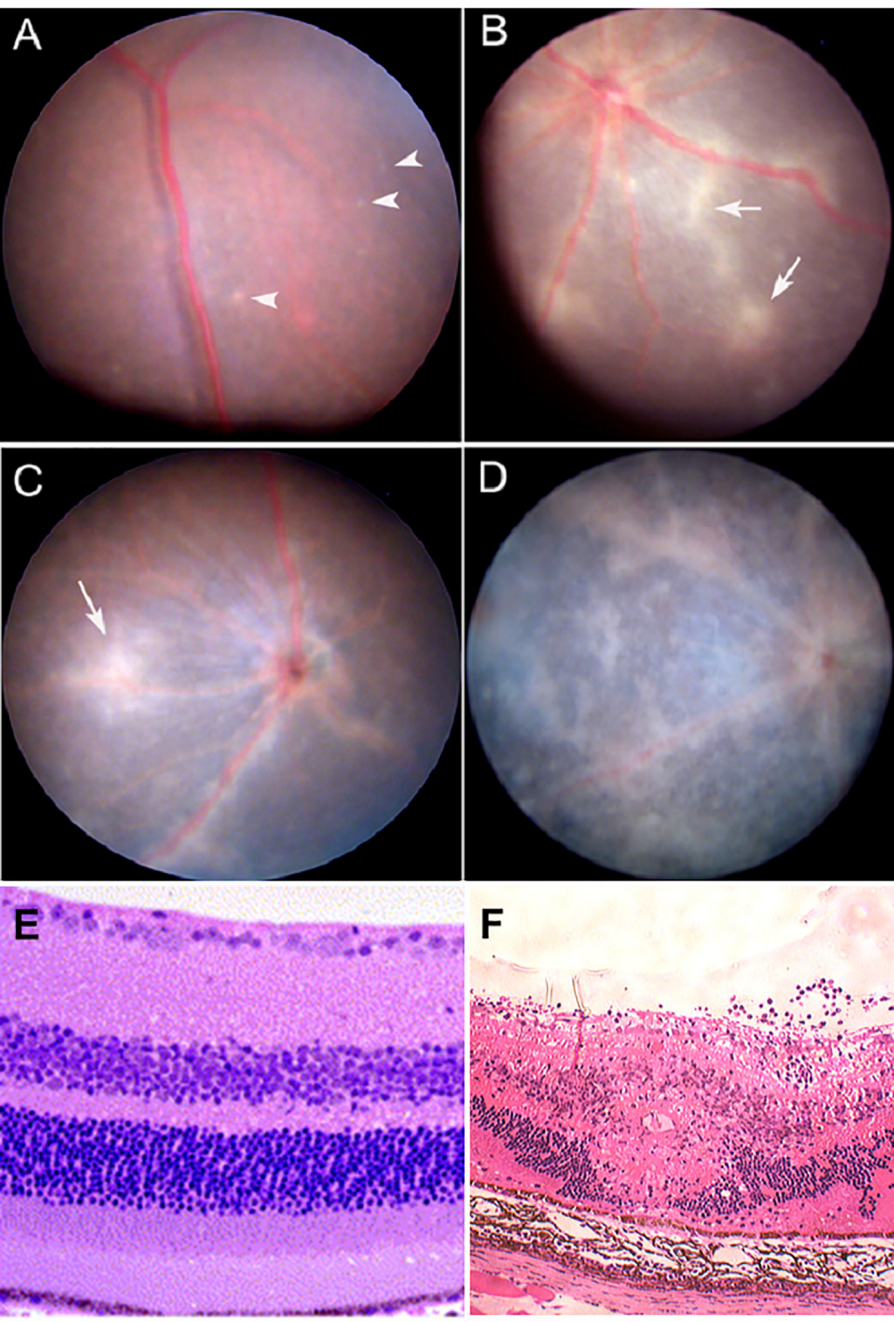
Experimental Autoimmune Uveoretinitis (EAU): a model for sight-threatening posterior uveitis. Clinical and histological signs of EAU in mice. EAU may be induced by subcutaneous injection of interphotoreceptor retinol binding protein (IRBP) peptide 161–180 in B10.RIII mice or peptide 1–20 in C57BL/6 mice with Complete Freund’s Adjuvant (CFA) and pertussis toxin (Ptx). Disease is mediated by a Th1/IL12 and/or a Th17/IL23 mechanism with dominant effect being Th17 mediated disease. The images are from the C57/BL6 mouse model. Clinical images are shown: **(A)** early focal chorioretinal infiltrate (arrowhead); **(B)** developing retinal vasculitis shown as focal perivascular “sheathing” (arrows); **(C)** extensive retinal vasculitis and large granulomatous infiltrate; **(D)** severe retinal inflammation, damage and atrophy; **(E)** Section of normal mouse retina; **(F)** EAU showing severe inflammation with large central area of retinal necrosis and loss of photoreceptors. Modified from (48) under copyright agreement with Elsevier Publishing license number 4887001207791.

**Figure 4:**
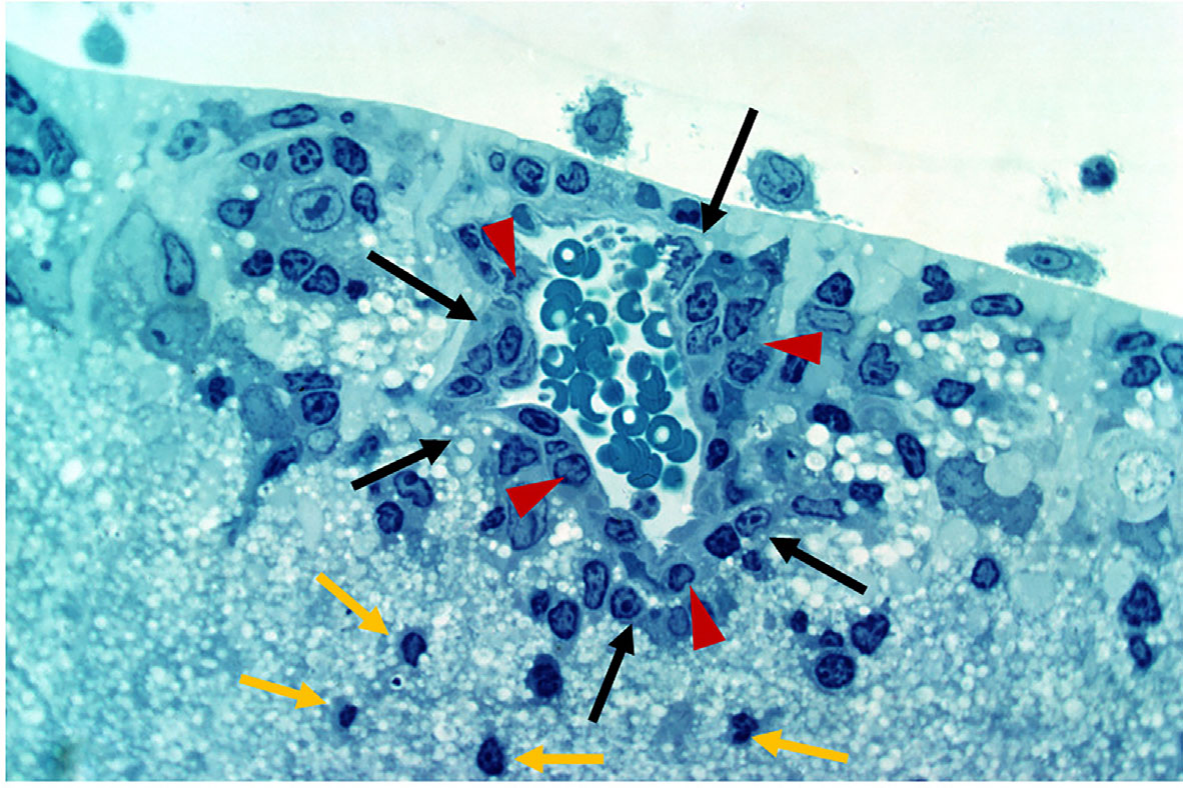
Experimental Autoimmune Uveoretinitis (EAU) in the rat model. EAU was induced in Lewis rats using retinal S antigen as described in (49). The image is a toluidine blue-stained thin section of the inner retina showing perivascular inflammatory cells (large arrows, black) retained within the glia limitans (arrow heads, red). Cells which have entered the retinal parenchyma have pyknotic nuclei and appear to be undergoing apoptosis (small arrows, yellow). Image provided by permission of the authors.

Defense against pathogen/antigen invasion is initially about barriers. Once the barrier is breached defense becomes a question of how quickly and effectively the pathogen can be cleared. The strength of the immune response is then the deciding factor and can be insufficient, sufficient, or exaggerated. In IP-high tissues, the barrier may be effective initially but under the right conditions, can be broken as in the classic model of experimental autoimmune uveoretinitis (EAU) (47, 48) (**Figures 3** and **4**). Damage can then be extensive and, in the case of the brain, life-threatening. However, many factors modify both the risk of BRB breakdown and the level of damage, particularly genetic factors; for instance, only a restricted number of rodent strains are susceptible to EAU (50, 51). Similar genetic susceptibility to BRB breakdown, as in uveoretinitis, occurs in humans (52, 53) (see below).

The skin and mucosal surfaces are probably the strongest barriers to pathogen invasion. However, these are not simply physical barriers but contain a wide array of immune cells and molecules which can kill or disable pathogens (54, 55). These entities include myeloid cells like Langerhans cells, dendritic cells (DC) and macrophages, a rich variety of T cells such as intraepithelial and dermal/stromal Teff and tissue resident memory (TRM) cells, mucosa-associated invariant T cells (MAIT), Treg, γδT cells, innate lymphocytes (ILC 1, 2, and 3) and NKT cells (56). Immune-modulating molecules include those constitutively present during homeostasis (defensins, neuropeptides, TGFβ, thrombospondin, and connective tissue activating factor/CTAF) (57) and cell associated ligand/receptor pairs such as Fas/FasL, Trail/DR5, CD47/SIRP-α, ICOS/ICOSL, and more (58, 59), as well as inducible molecules such as cytokines and chemokines. These activate resident immune cells as well as initiate a systemic immune response with recruitment of bone marrow derived cells to wall off and insulate against pathogen spread.

These external barriers are strong and display a level of IP whereby they quench potentially damaging immune responses (60). This is particularly important for the skin and mucosal surfaces, specifically the gut mucosa, since they are exposed to a vast panoply (trillions) of commensal microorganisms in the microbiome. This includes bacteria, viruses, fungi, archaea, and protozoa. In the gut, the microbiome actively participates in barrier homeostasis by secreting anti-inflammatory molecules such as short-chain fatty acids (SCFA), small molecules which can pass through the gut barrier into the system and influence immune cell function (61). In this regard, the intestinal and presumably other mucosal barriers share properties with CNS barriers which promote T cell tolerance and the generation of Treg cells (see below) [reviewed in (60)]. The function of the microbiome is not only to suppress settlement of pathogenic entities, but also to harvest and generate nutrients/metabolites (e.g. folate) and energy (e.g. SCFA) from nutritional sources which the host has to provide (62). SCFA have direct stabilizing effects on intestinal integrity (63) as well as priming effects on antigen APC and Treg (64–66) and appear to harness the protective function of gut ILC3 cells through free fatty acid receptor 2 (FFAR2) and the production of IL22 (67).

The skin and mucosal barriers to the external world thus have similarities to the internal barriers between solid organs and the blood. These barriers are variably complete, being easily permeated in tissues with “leaky” blood vessels such as liver and spleen, choroid plexus and uveal tract, and highly restrictive in the CNS. Here the NVU provides a complex physical, molecular, cellular and immunological barrier, in which pericytes, glia, perivascular macrophages and microglia collaborate (**Figure 2**). The microbiome has a significant role to play in the development of the BBB and perhaps also in the BRB [discussed in (68)]. Mice reared in germ-free conditions have increased permeability of the BBB, continuing on into adult life and appear to have reductions in tight junction proteins including occludin and claudin 5 (13), indicating a role for the microbiome in maturation of the endothelial tight junctions. Similar control may also be exerted by the microbiome over the development of the blood-testis barrier (69). We have mentioned that maturation of CNS barriers occurs during the first months/year of life under the control of the developing microbiome and disruption of this critical process in the neonatal period renders infants susceptible to infections, particularly common viral infections such as herpes simplex (HSV) and cytomegalovirus (CMV). It is at this time that lifelong viral latency is frequently established [for review see (11)]. Remarkably, these effects can be modified *in utero* since stabilizing the maternal microbiome can have beneficial effects on weanling BBB tight junction formation promoting reduced permeability (70). It is clear therefore that the founding of a healthy microbiome in the early neonatal period is necessary for the establishment of the IP status of the CNS in adulthood. There are many factors which influence the establishment of a healthy microbiome in this critical perinatal period such as the type of delivery, the use of antibiotics, and breast- vs. bottle feeding, the significance of which is now being fully appreciated (71).

Interestingly, at the opposite end of the aging spectrum, defects in the integrity of the NVU of the BBB are considered to underly neurodegenerative disease (72) and may be linked to conditions such as diabetes (73). Similar defects in the BRB are linked to age-related disease in the eye which have an underlying inflammatory pathogenesis (74, 75). Some of these conditions are proposed to be driven *via* a gut-CNS axis such as Alzheimer’s disease (76). The nature of the CNS immune response (“privilege”) may make it more susceptible to latent infections (see **Figure 1**) with pathogens such as CMV acquired in childhood and prions acquired in adulthood liable to reactivation as immune defenses weaken in old age [reviewed in (11)].

## Uveitis, Immune Privilege, and the Blood Retinal Barrier

The above considerations allow an interpretation of ocular inflammation, in particular uveitis, in the context of IP. Uveitis has been classified anatomically (anterior vs. posterior), etiologically (infectious vs. non-infections) and pathogenetically (autoimmune vs. autoinflammatory), most recently as part of the standardization of uveitis nomenclature (SUN) project (77) and has been reviewed elsewhere (68, 77, 78). Since the eye is composed of a range of discrete tissue types, inflammation may involve one or more of these tissues individually or together and “uveitis” (i.e. swelling and leakage of uveal blood vessels) is necessarily part of these. Thus, inflammation of the parenchyma of the ocular coats (scleritis, keratitis, iritis, cyclitis, and choroiditis) always involves inflammation of the uveal tract and its blood vessels. Inflammation at these sites does not usually involve the retinal vasculature. In contrast, inflammation of the retina (retinitis, retinal vasculitis) almost always involves uveal vessels. Acute AU is a self-limiting disease in which the retina is not usually involved. Anterior and intermediate uveitis may be complicated by cytokine-mediated secondary macular edema (swelling of the retina at its thinnest central region) in a bystander fashion if these conditions persist and become chronic but the retina is not usually directly under attack.

Thus, one can view AU, and associated conditions such as keratouveitis and sclerouveitis as ocular inflammatory conditions which do not break high level-IP since they do not breach the BRB. In this sense, the level of IP (see above comments on relative IP) expressed by tissues such as the cornea and sclera is not significantly different from other tissues which sit behind the external barriers of the skin and mucosa but outside the highly developed CNS barriers. Similar considerations apply to the coverings/surrounding tissues of the brain, i.e. the meninges. A possible “protective” role for IP can thus be considered specifically for CNS neural tissue (retina and brain parenchyma) but much less so for ocular and brain tissues outside the CNS barrier such as the uvea and meninges, and this is reflected in the much greater frequency of AU over PU (posterior uveitis) and meningitis over encephalomyelitis (11).

When IP is lost and the BRB and the BBB are breached, the outcome is sight-threatening and life-threatening, respectively. The most dangerous time is during development of the BBB and BRB in the prenatal and neonatal periods and in early infancy/childhood (see above section). Many infections acquired at this time are cleared only partially by the developing immune system; where tissues have lower levels of IP, infections are cleared quite efficiently and completely e.g. from the gut and its secondary lymphoid organs. However, from areas with higher levels of IP such as the CNS, the testes/ovaries and stem cell niches in the bone marrow, organisms which have made it through both the external and internal barriers, may further respond to the unfavorable CNS environment by becoming latent [reviewed in (11)]. Here, the immunological component of the IP-dependent CNS barrier comes into play in which Treg sustain latency by suppressing potential Teff and TRM cells, thereby preventing damaging immune responses in essential tissues and organs. The strongest evidence that this mechanism is functioning in immune defense is the “experiment of nature” brought on by the AIDS epidemic. Only when the global circulating T cell numbers, which includes Teff, TRM and Treg cells decline below 200 cells/ml and especially when they are less than 50 cells/ml, do latent infections, including those acquired in early life, become active infections and spread through the tissues (79, 80). This is particularly so for viral infections, such as CMV, HSV and varicella zoster virus (VZV) but also for infections acquired in adulthood which were held in check by the immune system such as *Mycobacterium tuberculosis* (Mtb) and *Toxoplasma gondii* (81–83). Even more convincing is that opportunistic infections by pathogens which would normally present no threat to the host, and in many cases would be regarded as “commensals,” come to the fore, particularly as brain and retinal infections, when T cell immunity in particular is compromised (e.g. pneumocystis) (84).

IP, therefore, as it affects the eye, is retina-centric and is primarily an immunological device whereby Treg and other immune regulatory cells, as major components of the BRB, control the activity of Teff cells. If engaged in clearing infection, Teff would generate excessive tissue damage. The gut microbiome not only plays a major role in development and maintenance of the physical BRB and its tight junctions but is central to the generation of the all-important Treg which underpin IP. When retina-centric IP breaks down retina-damaging sight-threatening uveoretinitis takes place.

In contrast, IP is relatively less important in non-retinal forms of ocular inflammation including autoimmune anterior uveitis (AAU) and corneal inflammation. Inflammation of the anterior segment is managed by the host in much the same way as inflammation at other sites including joints, muscle, and connective tissues generally. Indeed, the most common form of AAU is that associated with SpA and its etiology is similar (16, 56). Many cases of presumed non-infectious uveitis have recently been shown to be linked to infection and may in fact be due to persistent/latent infections delivered to the eye and other tissues *via* latently infected bone marrow precursors (85–88).

## Pathogenesis of Uveitis

The pathogenesis of uveitis can be considered by defining the conditions in which the disease occurs. Firstly, uveitis is a blanket term which applies to all forms of ocular inflammation involving any of the coats of the eye (**Table 1**). In infectious uveitis, where a replicating pathogen has been identified, the etiology is quite clear. In this context, endophthalmitis, including post-surgical endophthalmitis caused by bacterial or fungal infection is a particular form of uveitis. HSV stromal keratitis (HSK) is another characteristic and relatively common form of inflammation in which significant cell infiltration in the anterior chamber of the eye can be observed and indeed the entire uveal tract may be involved as has been observed in mouse models (46, 89). Scleritis caused by infection, such as Mtb produces a sclero-choroiditis which similarly involves the uveal tract, preferentially locating in the choroid (90, 91). As indicated in the section above, infectious retinitis in immunocompromised individuals invariably involves the uveal tract but the reverse is much less common, i.e. most forms of uveitis do not involve the retina. Infectious AU and PU can thus be defined quite clearly by the BRB.

**Table 1:** Terminology of ocular inflammation in uveitis.

Terminology of ocular inflammation (uveitis)		
Type of uveitis	Etiology	Tissues affected
Anterior	Infectious Non-infectious	Iris, ciliary body, sclera, cornea
Intermediate	Infectious Non-infectious	Ciliary body, vitreous, peripheral retina
Posterior	Infectious Non-infectious	Retina, retinal vessels, optic nerve

Disease classification is based on type, etiology and affected tissues in uveitis.

### Terminology of Ocular inflammation (Uveitis)

In the absence of infection, the etiology of ocular inflammation is less clear. Autoimmunity as a mechanism has been championed for many decades of the 20^th^ century, initially focusing on the uveal tract as a source of antigen [reviewed in (92, 93)]. The discovery of retinal antigens as potent inducers of PU (uveoretintitis, EAU) (94, 95) in animal models, similar in pathogenesis to other models of autoimmunity (experimental autoimmune encephalomyelitis, EAE; experimental autoimmune thyroiditis, EAT) (96–98) opened the door to a rich field of discovery relating to the pathogenic antigen-specific T cells (Th1, Th17), antigen presenting DC, effector macrophages and other myeloid cells as well as pro-inflammatory cytokines (TNFα, IL1, IL12, IL23) all participating one way or another with the mechanism of uveitis [reviewed in (99)]. Autoantigens in AU have been much more difficult to identify: perhaps the best model is the proteoglycan transgenic mouse in which there is a predominance of T cells expressing a T cell receptor (TCR) specific for the arthritogenic epitope of the G1 domain of the proteoglycan aggrecan (100). These mice develop a joint disease resembling spondylo-arthritis. A proportion of the mice also develop AU but, importantly, the uveitis does not involve the retina, once more emphasizing the divide between AU and PU, buttressed by the BRB.

However, evidence for autoimmunity in human uveitis, either anterior or posterior, is thin. While adaptive immune B and T cell reactivity to ocular antigens has been amply documented in patients with uveitis, similar activity has been reported in healthy individuals without disease [reviewed in (68)], and pathogenicity has been impossible to prove so far. Probably the best evidence might come from interventions such as cellular therapies (antigen-primed DC or Treg cell therapy) to demonstrate antigen specificity conclusively [reviewed in (101)].

Dysregulated innate immunity, or autoinflammatory disease, has more recently been proposed as a mechanism for many forms of uveitis. Endotoxic uveitis is a standard model in which local or systemic inoculation of endotoxin induces a short-lived ocular inflammation [reviewed in (102)]. Peptidoglycan and other pathogen-associated molecular patterns (PAMPs) such as CpG induce a similar type of inflammation, acting through pathogen recognition receptors including toll-like receptors (TLR) and C-type lectins and are mediated by downstream signaling pathways such as PI3 Kinase/Akt/NFkB, Syk-CARD9, and the Asc/NLRP3/inflammasome pathways (103–106). Most of these models resemble AU and do not involve the retina except in a bystander fashion. Similarly, uveitis associated with many of the human autoinflammatory syndromes and proposed autoinflammatory diseases such as SpA, is almost exclusively restricted to AU [reviewed in (68)]. The main exception to this is Behçet’s disease (BD), which can include AU, PU or panuveitis in its manifestations, as well as neuro-Behçet’s symptoms (cerebral vasculitis) (107–110).

Interestingly, recent studies have linked EAU, a recognized experimental autoimmune disease induced by specific antigen (50, 95), with gut microbiota. IRBP-TCR transgenic B10.RIII mice with a high precursor frequency (~25%) of T cells specific for peptide IRBP_161–180_ of the retinal antigen interphotoreceptor retinol binding protein (IRBP) develop spontaneous uveoretinitis gradually from p20 (post-natal day 20) and reaching an incidence of 100% by 2 months of age (111). However, disease severity is reduced if the mice are reared in a germ free environment (112). Similarly, C57BL/6 mice immunized with IRBP peptide IRBP_1–20_ emulsified in Complete Freund’s adjuvant (CFA) develop EAU but not in a germ free environment (113). Furthermore, treatment of mice with antibiotics prior to induction of EAU prevents disease but cannot stop disease once it has been induced (113). These data indicate that the microbiome strongly influences the risk of BRB breakdown and the immunological component of so-called IP depends on the condition of the microbiome. The question which remains is whether particular microbiota/commensals which constitute the microbiome specifically annul the protection of CNS IP. Studies in humans indicate that certain diseases such as BD and ankylosing spondylitis (AS) are associated with specific microbiome composition (16, 109, 110), although, if the above dependence of IP on the BRB is to hold true, retinal vasculitis in BD but not AU in AS might expect to be covered by IP.

A case has been made that retinal antigens are sequestered behind the BRB (114) and are not recognized by the peripheral immune system (immunological ignorance). In this scenario, a healthy BRB prevents access by autoreactive T cells to the retina and so, retinal inflammation is prevented. However, in the presence of dysbiosis commensal antigen-specific T cells are activated and circulate systemically. It is suggested that such T cells are cross-reactive with retinal antigens and have the potential to cross the BRB and initiate inflammation. How activated T cells cross the BRB is not clear, but it is well established that non-activated, naϊve T cells do not enter the tissues. The question is whether all activated T cells can enter the tissue. Prevailing dogmata support a pathogenesis which depends on precursor frequency and antigen dosage/availability, both of which determine whether, stochastically, such cells will become pathogenic (115–118). Most recently, in a neoantigen transgenic model of spontaneous uveo-retinitis, an absolute requirement for antigen-specific T cell activation has been demonstrated while non-specifically TCR-activated T cells fail to induce disease (119). These questions have exercised researchers over many years and the concept that cross-reactive T cells, activated by a dysbiotic microbiome, might be the guilty party adds significantly to pathogenetic possibilities. Candidate mechanisms include bystander damage, molecular mimicry, dual TCR expression and TCR promiscuity due to variable TCR-peptide MHC affinity. In addition, a possible innate immune, microbiome-based adjuvant effect has been proposed. In the case of the retina and EAU some of these questions have been addressed. In a transgenic model in which the foreign antigen Hen Egg Lysozyme (HEL) has been expressed in the retina under control of the promoter for IRBP (single transgenic IRBP-HEL, sTg : IRBP-HEL mice), adoptive transfer of HEL-TCR specific (3A9) T cells failed to induce EAU, even when the mice were co-infected with cytomegalovirus (MCMV) to induce a proinflammatory background microenvironment. However, when the mice were co-infected with MCMV which had been genetically modified to express the pathogenic HEL_41–60_ peptide, EAU developed within 7 days of adoptive transfer of 3A9 cells and the severity of uveitis depended on the level of HEL expression in the retina (41). T cell cross reactivity/molecular mimicry between retinal and viral antigen therefore appeared to be the dominant mechanism generating pathogenicity, while bystander activation was essentially discounted. These data indicate that T cell activation by specific antigen underpins their pathogenicity in this model of autoimmune disease. Interestingly, a recent study has shown that chimeric human-viral proteins can be generated during viral infection in humans which may offer a further novel explanation for how infection and autoimmunity may overlap (120).

The other side of the microbiome coin is the abundant evidence that a healthy microbiome maintains a proportionate peripheral Treg population through mediators such as SCFA and folate (see below), and Treg are known to be central to the maintenance of immune tolerance and the prevention of autoimmunity (121) (see below). In addition, there is accruing data from human studies that autoimmune/autoinflammatory uveitis syndromes such as Vogt-Koyanagi-Harada disease (VKH) and BD are associated with unsuspected dysbiosis (109, 122) and the failure/lack of Treg cells.

The concept that the gut might drive autoimmune disease in the eye, as has been shown for gut-associated segmented filamentous bacteria in the joint (123), deserves careful examination in relation to IP. Disturbed microbiota has been linked to numerous non ocular as well as ocular inflammatory conditions [reviewed in (54)] and a direct relationship to gut-derived T cells, cross reactive with tissue antigens has not been reported in other models. In addition, a link between a dysregulated microbiome and systemic autoimmune/autoinflammatory disease is not restricted to conditions associated with disrupted IP. Thus, if the microbiome is implicated in the pathogenesis of uveitis, this would appear to be irrespective of IP status, thus relegating IP to a lesser, if any, role. While microbiome-based antigens are currently being evaluated for a role in ocular inflammatory disease, the precise relationship is likely to be complex.

## Role of Treg and the Microbiome in Immune Regulation/Prevention of Uveitis

The gastrointestinal tract is now recognized as a major immune organ, containing an abundance of innate and adaptive immune cells which influence immune responses locally and at distant sites (124). The microbiome with its superabundance of micro-organisms directs the development of T cell populations particularly Th17 cells and Treg, responsive in an antigen-specific manner to microbial antigens. The innate immune system responds in an antigen non-specific manner to produce anti-microbial peptides such as α- and β-defensins in response to cytokines such as IL22, IL18, and INFγ (125). From the moment of birth and even prenatally, microbial colonization shapes the developing immune system and reciprocal interactions benefit (or otherwise) the development of a healthy immune system. The influence of the microbiome on IP therefore relates to several of the components of the blood-CNS barrier: in addition to guiding maturation of tight junctions between BBB endothelial cells (13), the microbiome induces the generation of commensal antigen-specific Treg (126–128), which not only act locally but have a specific gut phenotype combining RORγt and FoxP3 transcription factors (129, 130), and are central to the prevention of intestinal inflammation and IBD (131, 132). In addition, they may have an extra-intestinal effect in the regulation of systemic autoimmune disease such as collagen-induced arthritis (133).

Treg are antigen-specific in their generation but not so in their effector function where they have broader regulatory properties (134). Importantly, the plasticity of Treg both in their generation and their activity is relevant to the above concepts of how commensal antigens might cross-react with tissue-specific antigens through molecular mimicry and induce uveitis (114). Peripheral Treg (pTreg) develop in the secondary lymphoid tissues, including the gut, from naїve T cells under the influence of their cytokine environment which includes both proinflammatory cytokines such as IL6 and regulatory cytokines such as TGFβ and IL22. Generation of Treg in the gut has been linked to the process of T cell education in the thymus although clearly there are differences (135). In the presence of antigen (here we are considering commensal antigen rather than self-antigen as in thymus) T cells may develop along a Teff pathway to generate Th17 cells and then undergo antigen-induced cell death (AICD). Alternatively, in the presence of folate such Teff either convert to homeostatic Th17 cells which generate IL22 (135), or they may become anergic (Tan) and continue on to convert to FoxP3^+^ Treg (136–138). Both thymus-like Helios^+^, neuropilin^+^ (natural, n) Treg and “peripheral,” induced (i) Helios^-^ Treg occur in the gut but iTreg are induced by the microbiome while nTreg are present from a pre-weaning stage (139–141). In addition, there are intraepithelial FoxP3^-^ Treg and CD4^+^ CD8αα^+^ Treg which have an important regulatory role to play (142, 143). However, if the milieu is not conducive, commensal antigen-generated Teff (Th17) cells may clonally expand and enter the circulation as activated cells, with the risk of causing disease if they meet cognate antigen (similar to thymic autoreactive T cells which have escaped deletion). Disease may then ensue where access to tissue antigen is possible and co-stimulation is available. However, for tissues behind “heavy duty” barriers such as the BRB and the BBB, where access may be dependent on a sufficient precursor frequency of activated Teff, released from the constraints of Treg, additional factors to antigen specificity are required. These may be delivered through upregulated innate immune responses (41) and in the case of dysbiotic commensal antigens, through an “adjuvant” effect (114).

In homeostasis, Treg induced by the microbiome promote tolerance rather than immunity. The beneficial/tolerance-inducing role of the microbiome and secreted bacterial products in this scenario is becoming better understood. Mention has already been made of the range of SCFA (propionate, acetate, and butyrate) and other bacterial products which apart from promoting epithelial integrity, promote Treg homeostasis [reviewed in (135)]. In addition, the labile, plastic Th17 cell which can develop into a Treg in the colon is induced by resident DC and other myeloid cells in the small intestine (144, 145). This, however, is a double-edged sword since some intestinal T cells possess dual TCR reactivity and under certain circumstances, can induce autoimmune disease in distant organs such as the lung (146). Dual TCR-expressing T cells are a recognized hazard for the development of autoimmunity by limiting the generation of self-reactive Treg, as has been shown in the non-obese diabetes-prone mouse (NOD) (147). This possible mechanism may also apply to autoimmune uveitis induced by commensal antigen-activated retina-specific T cells although the evidence currenlty appears to be against this notion (114).

## Genetic Susceptibility to Uveitis in the Context of the Microbiome and Treg: ASSOCIATION Between Dysbiosis and Uveitis

The genetics of uveitis also raises questions about the role of IP in conditions in which uveitis features. IP as a phenomenon was hypothesized during the time when MHC antigens were being discovered and was based on failure of the host to react to alloantigens transplanted to the anterior chamber of the eye. Since then the importance of MHC antigen has been revealed particularly in corneal graft rejection (148–150) suggesting that MHC antigens themselves were not the IP-responsive antigens but that the host responded to some tissue allografts through alternative limited polymorphisms. This undermines the original notion of IP as it was conceived but supports the concept of tissues modulating the host response to foreign antigens including alloantigens. To extend Matzinger and Kamala’s concept of tissues in control of the immune response (2), not only do different tissues such as the skin, gut, adrenal gland and retina modify the immune response in different ways, but each individual minor polymorphism in each tissue’s proteins will have an influence on the host response. This is borne out by the variable pattern of disease found in different individuals and contributes to the overall variability, exemplified by the current Corona virus (Sars-CoV-2) pandemic in which the upper respiratory tract and the lungs are the main target of attack but the individual outcome varies from fatal Covid-19 disease to severe cases with long-lasting effects [individuals referred to as “long-haulers” (151)] to no symptoms depending on the severity of the associated host immune response (152, 153). Large scale “big data studies” performed through consortia such as *The Infection and Immunity Immunophenotyping* (3i) consortium have revealed the influence of a large number of immunoregulatory genes (>140) on the properties of different tissues underpinning immune variation which resonate with a much broader interpretation of how different cells and tissues influence the outcomes of immune challenge (154), and the future invites further similar study to reveal how CNS tissues including the eye fit with these paradigms.

Both human uveitis and experimental models of uveitis have quite specific and wide-ranging genetic susceptibilities. Caspi’s initial studies in mice confirmed the importance of minor antigens in murine susceptibility to induction of EAU (51). Further studies defined the roles of a range of innate immune genes such as TLR, C-type lectins, and genes regulating the inflammasome and IL1 production (103, 155–158). Indeed, an essential role for IL1 in EAU induction has been shown (159).

In human uveitis, strong genetic links have been established with certain types of uveitis. The long known association of the MHC antigen HLA-B27 with AU has been extended with more recent information on susceptibility links to antigen processing genes such as endoplasmic reticulum peptidase 1 (ERAP1) but also to genes more closely associated with innate immunity such as nitric oxide synthase 2 (NOS2) as well as less strong links to a number of other proteins such as Mer Proto-oncogene tyrosine kinase (MERTK), kinesin-associated protein 3 (KAP3) and acetyl-Coenzyme A acyltransferase 2 (ACAA2) (160) The strongest association of MHC antigens and any human disease is that between HLA-A29 and the rare uveitis condition birdshot chorioretinopathy (BCR) (161). Further linkages with BCR have been found with functionally distinct ERAP-1 and -2 genes in a large population of BCR patients (162). BD has linkage with HLA-B51 while sympathetic ophthalmia and VKH disease have common associations with HLA-DR0405 and, interestingly, T cells from patients with VKH show cross-reactivity with peptides from CMV and the melanocyte protein tyrosinase (163), once more implicating infection in a well-defined autoimmune disease.

In many of these conditions, shared linkages with innate immune response (IR) genes, such as the IL23 receptor (164) and cytokine genes of myeloid cells (52, 165–167) have been observed. However, most of the associations are particular to specific disease entities and no clear pattern emerges for adaptive or IR genes for ocular inflammation as a whole, whether it be retina-centric, i.e. behind the BRB or not. No MHC or non-MHC association has been described for IP *per se*. IP was originally attributed to a lack of MHC expression in the relevant tissues (cornea, brain parenchyma, retina, hair follicle, testis etc.) which, in terms of MHC Class I and II still has some relevance due to the paucity of overall expression in these tissues. However, an analysis of CD11c expression on myeloid cells in brain vs. other tissues revealed a significant down regulation in CNS myeloid cells, indicating that while such cells were present in the tissues, the microenvironment of the tissue had the greater influence on whether IR genes were likely to be activated (168).

In contrast to genetic associations with IP, clear connections with some types of uveitis and a disturbed microbiome have been described. For instance, VKH (122), HLA-B27-associated AU [reviewed in (169)], and uveitis in BD (110) have all been linked to dysregulated microbiota with *Prevotella* sp. being identified as a consensus bacterial community in which further stratification linked *Clostridiales* sp. to neuro-BD and *Bacteroides* with multiple sclerosis (170). Importantly, Treg which are considered to play a role in maintaining IP in the retina (43, 44, 171, 172) and brain (173–175) have demonstrated clear tissue adaptation at the barrier sites of the skin and the intestine (176) and it is likely that they do so at sites of the BRB and BBB. Thus, in terms of uveitis there is no clear pattern of disease susceptibility. Indeed, the range of genetic susceptibilities based on MHC Class I and II, and minor/tissue antigen polymorphisms do not support the concept of a unique property of IP for any particular tissue. Tissues’ abilities to deal with pathogen invasion and foreign antigens depend on the tissue as well as the genetic make-up of the individual, but probably more importantly it depends on the tissue/cell tropism and virulence of the pathogen.

## Restoring Microbiome-Associated Gut Immune Privilege to Prevent and Treat Uveitis

As indicated above, IP, when considered as a process whereby tissues modulate immune responses, is by definition a property of most if not all tissues and the gut is no exception (60, 177). Commensal microbial and dietary antigens are “tolerated” in a privileged manner in the gut, mediated by a unique set of Treg (130, 139) which exert control over potential Teff cells. In this, they are assisted by a range of innate immune lymphocytes (124), “tolerant” DC and specialized T cells such as γδT cells and CD8αα^+^ mucosal epithelial T cells [reviewed in (178)]. As discussed above, disturbance of the gut microbiota (i.e. dysbiosis) is linked to disease, particularly IBD but is also implicated in various autoimmune diseases such as rheumatoid arthritis, type 1 diabetes mellitus, multiple sclerosis and autoimmune liver disease [reviewed in (179)]. Caspi’s group has shown that spontaneous EAU in IRBP-TCR transgenic mice can be prevented and/or suppressed in microbiota-deficient mice, as well as in mice treated with an antibiotic cocktail and has proposed that retina-specific T cells are activated by microbiome-derived antigens which escape through a “leaky gut” epithelium and are presented to T cells by gut or mesenteric lymph node DC (112). The activated T cells then enter the circulation and cross the BRB where they connect with cognate antigen (IRBP) and become pathogenic. Precisely why retina-specific T cells should become activated by microbiota-associated antigen is not clear, but several mechanisms have been proposed. Bystander activation is unlikely but molecular mimicry/antigen cross-reactivity are genuine possibilities (41), while dual TCR expression discussed above is also an interesting option (147) although unlikely as argued by Horai and Caspi (114). Hybrid self-foreign antigen is a novel paradigm (120). Similar studies in the IRBP-inducible model of uveitis have also been reported, showing that EAU can be suppressed in antibiotic-treated mice (180) while IRBP-induced EAU is completely suppressed in germ-free mice (113). In this model, the effect of antibiotic administration is essentially preventive and not therapeutic, i.e. once the disease is active, antibiotic treatment is ineffective (113). These studies, in both the spontaneous and inducible models of EAU, implicate an underlying dysbiosis which permits translocation of bacterial antigen across the lining epithelium, and indeed this is the case. In CFA/IRBP-induced EAU where an antigenic peptide (IRBP) and CFA-mixed emulsion is inoculated subcutaneously, defects in the gut epithelium have been demonstrated with inflammatory cell infiltration in the gut wall (181). Interestingly, these effects on the bowel did not seem to be IRBP-specific since similar findings were observed with CFA-mycobacterial antigen alone. In addition, the numbers of circulating IRBP-specific T cells (Teff or Treg) when CFA/IRBP was inoculated were vanishingly low while the numbers of microbial antigen-specific cells were significantly higher, as might be expected. However, an associated dysbiosis may not be required for microbiota-antigen reactive/activated cells to be present in the circulation. Hegazy et al (182). have shown that in healthy humans, microbiota-specific activated CD4 T cells are present in the blood, and are responsive to a range of commensal bacteria. In patients with IBD, the frequency and specificity of these T cells is increased (182).

## What Does the Gut–Central Nervous System Axis Imply for Therapeutic Intervention?

Several groups suggest that there are significant opportunities to intervene, particularly by promoting Treg induction in the gut to eliminate dysbiosis and restore a healthy microbiome. Commensal bacteria provide many mediators of immune and metabolic homeostasis including SCFA. Attempts to ameliorate autoimmune disease by administration of SCFA have been quite successful experimentally in models of multiple sclerosis, hepatitis and diabetes (109, 183–187) as well as uveitis (188). The mechanism of action appears to be through induction of Treg which act locally in the gut as well as at a distance on barrier structures such as the BRB (189–191).

Bacteria produce other metabolites and nutrients. These include bacterial metabolites derived from bile acids, tryptophan, indole and activators of the aryl hydrocarbon receptor (AhR) and folic acid (FA). The latter is of particular interest since it has a direct impact on the survival of intestinal Treg (65) predominantly mediated *via* one of the folate receptors (FR4). FA deficiency is intrinsically damaging to the eye and has been linked to amblyopia (192), optic neuropathy (193), senile cataracts (194), and retinal hyper-homocysteinemia (195) along with its secondary complications (196–198). Hyper-homocysteinemia is connected to specific polymorphisms in FA metabolism-associated genes (199) and has been reported to occur in autoinflammatory BD patients (200). A role for FA in autoimmune uveitis is therefore recognized [for review see (201)].

The direct effects of FA on the eye are delivered *via* several routes including folate receptors (FR; folate-binding proteins) (202), the reduced folate carrier, and the proton-coupled folate transporter (203). FA transport proteins are ubiquitously expressed, and are abundant in retinal pigment epithelium (RPE) and retina (204), suggesting a potential direct immune-modulating role for folate at these sites, particularly as the immunoregulatory BRB cell (the RPE) has the potential to convert naïve T cells to Treg. In humans, there are four known FR isoforms (α, β, γ, and δ) with tissue-specific expression patterns (202, 205). Most recently the human receptor homolog for murine FRδ (also known as FR4 or folate binding protein 3) has been found most abundantly expressed on Treg cells (206). In mice (and possibly in humans), FR4 is expressed under the control of the transcription factor FoxP3 (206), whose sustained expression is a key factor in Treg functional stability (206–210). FoxP3^+^ Treg populations in the colon are sustained by oral FA supplementation; in contrast, a diet deficient in folate leads to a marked reduction of FoxP3^+^ Treg selectively in the colon and increased autoimmune bowel inflammation.

The induction of Treg in the periphery, including the gut, is a major control mechanism in the resolving stages of inflammation. Peripheral Treg are generated from naїve T cells but may also be derived from anergic T effector cells (Tan) through a reciprocal induction process (211). T cell anergy is a major contributor to peripheral tolerance mechanisms, wherein CD4^+^ T cells lose the capacity to produce autocrine growth factor and proliferate in response to antigen (212, 213). A subset of FoxP3^-^ CD44^hi^ CD73^hi^ FR4^hi^ anergic CD4^+^ T cells has been identified (214) which upon adoptive transfer, gave rise to Treg cells in an autoimmune arthritis model (211), thereby functioning as progenitors for Treg cell differentiation following TCR-mediated anergy reversal. It has been hypothesized that upon recurrent antigen encounter, anergic CD4^+^ FoxP3^-^ FR4^+^ CD73^+^ Nrp1^+^ T cells are prone to partial de-methylation in a specific signature gene, generating ideal progenitors for the peripheral differentiation of stable FoxP3^+^ Treg (211, 215). These anergy-derived Treg suppress immunopathology and reinforce anergy induction (215). In this context, we have recently shown, in a transgenic model of spontaneous uveitis due to failure of Treg induction, that Tan convert to Treg cells as the inflammation resolves (119). Furthermore, adoptive transfer of antigen-experienced Treg completely prevented development of this spontaneous disease, showing the importance of Treg in the control of uveoretinitis. In the development of cell therapies, such as Treg, for autoimmune disease it would seem there should be a role for FA.

Alternatively, FA may be included in the proposed use of probiotics for control of autoimmune disease (216). Probiotics are preparations which deliver bacteria beneficial to the host through mechanisms such as improving gut epithelial integrity and promoting a balanced immune system. In contrast, prebiotics such as dietary fiber feed the gut microbiome. Probiotics act by becoming part of the commensal community of the microbiome and thus may promote intestinal Treg generation. Some probiotics such as those containing *Lactobacillus (L.) reuteri* WH1689 (217) and *Streptococcus gallolyticus subsp. macedonicus (S. macedonicus)* CRL415 (218), have genes conferring specific beneficial properties affecting metabolism and FA biosynthesis. Recently, probiotic *L. reuteri* has been shown to convert CD4 T cells into CD4^+^ CD8αα^+^ double positive intraepithelial cells, which as indicated above are immunoregulatory cells, by decreasing the transcription factor THPOK through activation of the AhR (219). Probiotics have been shown to be effective in certain autoimmune conditions [reviewed in (220)] and are part of what has been termed “the global preclinical pipeline” which includes small molecules such as SCFA as well as engineered probiotics and phages (221). Both probiotics and prebiotics (such as glucans and fructans) are under intense investigation as regulators of general health and homeostasis (222).

Despite the avalanche of studies and projects evaluating probiotics, there is still continuing uncertainty about their overall effectiveness in the context of active autoimmune disease. For this reason, preclinical studies are important. Heat-killed *L. reuteri GMNL-*263 was found to be effective in preventing cardiac damage in a model of systemic lupus erythematosus (223); in a second study, a commercially prepared probiotic, *Lactibiane Iki*, controlled active EAE by promoting tolerogenic DC (224); and the combined administration of two *Bifidobacteria* and *Lactobacilli* probiotic strains prevented experimental myasthenia gravis (225). Preclinical studies of probiotics are in progress but to date no randomized controlled trials of probiotics in patients with uveitis have been reported. However, the opportunities for such studies are becoming clearer (226).

## Conclusion

The concept of IP in the context of uveitis is difficult to sustain. The pathogenesis of uveitis (whether it be infectious or “non-infectious”) (68, 78, 114, 227) is different depending on whether the inflammation occurs in front of or behind the BRB. Even if the site of inflammation is in a tissue which presents a greater immunomodulatory microenvironment, as in the retina compared to the iris, the classical IP properties of the tissue (such as immunosuppressive mediators and lack of resident MHC Class II^+^ DC) are not overly effective in preventing inflammatory disease (uveoretinitis). Perhaps a fresh look at what IP as an immunological concept entails is required, specifically asking the question: is IP intrinsically different from mainstream mechanisms which deliver immunological tolerance? Relative degrees of IP can be applied essentially to all tissues and are very highly developed in healthy tissues such as the skin and the gut where a multitude of immunoregulatory mechanisms and cell types are positioned to control the immunostimulatory potential of vast colonies of commensal bacteria. Breakdown of these protective shields is the initial stage of development of systemic and organ-specific inflammatory diseases. This is dependent on the cross reactivity (or other mechanisms as discussed above) of microbiome/commensal pathogenic antigens to activate and sufficiently expand tissue-specific T cells which can cross internal barriers and cause disease on encountering cognate antigen. Therapies directed toward generation of regulatory cells (Treg or other) which will restore immune homeostasis, particularly in the bowel, may be the way forward to restoring the IP status of all tissues, including those of the CNS.

## Author Contributions

CM researched, collated, and summarized available literature, drafted the article, and wrote parts of it. JVF conceptualized and wrote the main body of the manuscript. CM edited and formatted the manuscript and figures. JH, HMW, and LK provided expert opinion, edited and critically revised the article. All authors contributed to the article and approved the submitted version.

## Funding

The ideas developed in this article were derived from work supported by Fight for Sight, The Eye Charity (CSO project grant award: 3031-3032) awarded to HMW, and by The Development Trust of the University of Aberdeen (Saving Sight in Grampian) (grant codes: RG-12663, and RG-14251).

## Conflict of Interest

The authors declare that the research was conducted in the absence of any commercial or financial relationships that could be construed as a potential conflict of interest.
